# Health hazard assessment and cooking effects on toxic metals in marine fish from the mediterranean sea at the Damietta Coast, Egypt

**DOI:** 10.1038/s41598-025-33257-3

**Published:** 2026-01-12

**Authors:** Amira Hegazi Makroum, Amira Ibrahim Zakaria, Hend Ali Elshebrawy, Khalid Ibrahim Sallam

**Affiliations:** Food Hygiene, Safety, and Technology Department, Faculty of Veterinary Medicine, Mansoura University, Mansoura, 35516 Egypt

**Keywords:** Arsenic, Mercury, Lead, Cadmium, Thinlip grey mullet, Sardines, Sand smelt, Health hazards, Environmental sciences, Risk factors

## Abstract

This study aimed to determine the toxic metal contents in thinlip grey mullet, sardines, and sand smelt, as well as the impact of cooking on the metal levels and their potential harmful effects on public health. Two hundred forty fish samples, comprising 80 each of the three identified species, were collected from the Mediterranean Sea at Damietta coast, Egypt and analyzed for arsenic, mercury, lead, and cadmium contents, which were detected at mean ± SE concentrations (mg/kg) of 2.29 ± 0.22, 0.119 ± 0.019, 0.651 ± 0.131, and 0.042 ± 0.007 in thinlip grey mullet; 1.68 ± 0.18, 0.098 ± 0.018, 1.011 ± 0.169, and 0.049 ± 0.008 in sardine; and 1.87 ± 0.16, 0.055 ± 0.057, 0.965 ± 0.186, and 0.052 ± 0.009 in sand smelt, respectively. Alarmingly, 100% of fish samples exceeded the permissible limit for As, while Hg levels were within the safe limits for all tested samples. Frying and grilling significantly reduced the metal levels. The target hazard quotient, total target hazard quotient in all tested fish samples, exceeded their approved limits for arsenic and lead, while the assessment of the cancer risk values for arsenic in all tested fish species surpassed the cancer slope factor, indicating potential public health risks associated with consuming such fish. This study highlights the need for strict control measures to limit contamination of aquatic resources, especially by arsenic and lead, and to ensure the safety of seafood for human consumption, protecting public health and reducing the risk of fish contamination.

## Introduction

Egypt is a major fish-producing country due to its unique geographical location. It is located in the northeastern corner of Africa and extends into Asia through the Sinai Peninsula, making it a transcontinental nation. To the north, Egypt has about 995 km of coastline along the Mediterranean Sea, while to the east, it has approximately 1941 km of coastline along the Red Sea, as well as several lakes, including Manzala, Burullus, Mariout, and Bardawil. Contamination of fish with high levels of toxic metals poses serious health risks to consumers. However, few studies are available on the levels of toxic metals in marine fish from the Egyptian Mediterranean coast.

Damietta hosts one of the largest fleets of Mediterranean fishing boats, accounting for half of Egypt’s fishing vessels^1^. It is situated northeast of the Nile Delta, between Alexandria and Port Said, with a 9-kilometer coastline along the Mediterranean Sea. Due to its strategic location, Damietta is a significant commercial port and a vital hub for Egypt’s fishing industry. Near the Damietta Strait, where the Nile meets the sea in Ras ElBar, lies Ezbet ElBorg, one of the most well-known fishing spots. This area is famous for various bony fish (Osteichthyes), including thinlip grey mullet, sardines, and sand smelt.

The thinlip grey mullet (*Chelon ramada* Risso, 1827) belongs to the Mugilidae family and is known in Egypt as “Tobara” or “Dubara.” It is an economically significant species, recognized for its ability to adapt to different salinity levels more than other mullet species^2^. This species is common in the coastal regions of the Egyptian Mediterranean Sea and inland waters^3^, and it has increasingly become a primary species in aquaculture to support and expand fish farming in Egypt^4^. Sardines (*Sardina pilchardus*; European pilchard) belong to the Clupeiformes family and are called “Al Sardine Al Baladi” in Egypt. The Sand smelt (*Atherina boyeri* Risso, 1810), part of the Atheriniformes family, is known as “Basaria” in Egypt. These three fish species are highly valuable food sources, rich in high-quality protein essential for muscle building and repair, as well as omega-3 polyunsaturated fatty acids that promote brain health, reduce inflammation, and protect against chronic diseases like heart disease. hey are also rich in B-complex vitamins, vitamins D and A—which contribute to immune function, bone development, and cellular growth—as well as essential minerals, including iodine (thyroid regulation), selenium (antioxidant defense), calcium (skeletal health), and iron and zinc (hematological and metabolic functions), making them valuable components of a balanced diet^5^.

Due to the excellent taste and high protein-to-fat ratio of fish compared to poultry and red meat^6^, along with its significant commercial value, global demand for fish is increasing as the population grows^7^. The average annual fish consumption in Egypt is approximately 20.84 kg per person, which constitutes about 57.096 g per day^8^. Despite these advantages, the fisheries sector in Egypt faces major challenges, including overfishing, climate change, and especially pollution, notably from toxic metals. Heavy metals are categorized into toxic metals such as arsenic, mercury, lead, and cadmium, and essential trace elements like zinc and iron^9^. Pollution levels in the Mediterranean waters near the coastlines have reached critical points^10^.

Toxic metals enter the marine environment via natural sources like volcanic eruptions, atmospheric deposition, and human activities such as industrial and agricultural processes and untreated sewage waste. These pollutants accumulate in fish and can move up the food chain to humans^11^. Various factors influence this accumulation, including water salinity, pollution levels, sediment characteristics, and available food^12^, along with water pH, temperature, fish species, size, age, sex, feeding behaviors, and length of metal exposure, all of which significantly impact metal build-up^13^.

Arsenic (As) is a naturally occurring metal, but is also prevalent due to human activities like mining, agriculture (fertilizers and pesticides), and various industries such as wood preservation. Mercury (Hg) is used in thermometers, pressure sensors, and dental applications, and mercuric chloride serves as a disinfectant; inorganic mercury is legally used in paints and cosmetics in the U.S. Lead (Pb) is found in batteries, plumbing, and gasoline additives, mainly lead carbonate. Cadmium (Cd) often appears as a by-product of copper, lead, or zinc smelting and is used in batteries and pigments^14^.

Toxic metals are characterized by their chemical stability, non-biodegradability, ability to bioaccumulate within cells, and extended half-lives^15^. These factors increase their levels in the food chain and enable their transmission to humans, leading to acute or chronic diseases, making their management a significant challenge^16^.

Cooking techniques improve the tenderness and flavor of fish, but they can also influence the levels of toxic metals, which may potentially impact the safety of the final product. Most studies focus on raw seafood to evaluate the public health risks associated with toxic metals; however, this does not accurately represent reality, as people typically consume cooked fish^17^. The different cooking processes can reduce toxic metal concentrations, rendering risk assessments based on raw food data unrealistic or inaccurate^18^.

Although thinlip grey mullet, sardines, and sand smelt are commonly consumed in Egypt, few studies have investigated toxic metal residues in these species and their potential toxic effects on public health. Therefore, the current study aimed to evaluate the contents of As, Hg, Pb, and Cd in thinlip grey mullet, sardines, and sand smelt harvested from the Mediterranean coast, as well as to assess the impact of grilling/frying on their metal concentrations. In addition to determining the non-carcinogenic and carcinogenic human risks associated with the consumption of such fish species by calculating Estimated Daily Intakes (EDIs), Target Hazard Quotients (THQs), Total Target Hazard Quotients (TTHQs), and Target Cancer Risk of tested metals.

Although thinlip grey mullet, sardines, and sand smelt are commonly consumed in Egypt, particularly along the Mediterranean coast, there is a notable lack of data on the concentrations of As, Hg, Pb, and Cd in these species caught from the Damietta region. Existing studies have primarily focused on raw fish or other geographic areas, and very few have looked at how conventional household cooking techniques like grilling and frying affect the amounts of harmful metals. Furthermore, human health risk assessments, including Estimated Daily Intakes (EDIs), Target Hazard Quotients (THQs), Total Target Hazard Quotients (TTHQs), and Target Cancer Risk, for these locally consumed species remain largely unavailable. Therefore, this study addresses these critical gaps by determining the levels of As, Hg, Pb, and Cd in raw and cooked thinlip grey mullet, sardines, and sand smelt from the Egyptian Mediterranean coast (Damietta area), evaluating the effects of grilling and frying on metal concentrations, and assessing associated non-carcinogenic and carcinogenic health risks for consumers.

## Materials and methods

### Study area

**Fig. 1:**
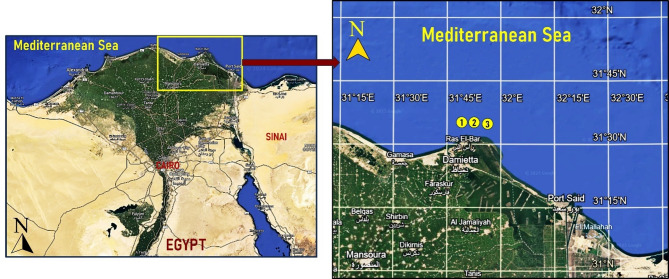
Map of the sampling area for the fish analyzed in the present study. Satellite basemap imagery was obtained from Google Earth Pro (Version 7.3.6; Google LLC; https://www.google.com/earth/*).* Labels, coordinates, and graphical elements were added using Microsoft PowerPoint (Version 2021; Microsoft Corporation). Imagery source: © 2025 Google, Maxar Technologies.

Ezbet El Borg is one of the largest fishing industries in Damietta Governorate, Egypt, boasting approximately 9 km along the Mediterranean Sea coastline. It lies on the northern coast of Egypt at the mouth of the Damietta River, which is a distributary of the Nile, directly opposite Ras El Bar. It is located 15 km northeast of Damietta and 210 km from Cairo. Fish were harvested from 3 different adjacent sites, 6–8 km from the Ras El Bar coast. The latitude and longitude coordinates of the sampling sites 1, 2, and 3 were 31°34′N,31°45′E→31.56°N, 31.75°E; 31°34′N, 31°46′E→31.566667°N, 31.766667°E; and 31°34′N, 31°47′E → 31.566667°N, 31.783333°E. The map of the sampling sites and the study area is shown in Fig. 1.

### Sample collection

A total of 240 fish, including 80 each of thinlip grey mullet (*Chelon ramada*), sardines (*Sardina pilchardus*), and sand smelt (*Atherina boyeri*), were harvested from the Mediterranean Sea at the Damietta coast between December 2024 and February 2025. The collected samples were humanely killed by percussive stunning with a heavy wooden handle. Samples were placed in an icebox and transferred to the laboratory, where they were subjected to weight and length measurements. The length and the body weight of thinlip grey mullet, sardines, and sand smelt are demonstrated in Table 1. Each sample was packed separately in clean polyethylene bags, labelled, and subjected to toxic metal analysis.

**Table 1:** Body weight and length of sampled fish.

Fish	Range and (mean ± SE) of body length (cm)	Range and (mean ± SE) of body weight (g)
Thinlip grey mullet	21–30 (25.3 ± 2.8)	135–282 (198.5 ± 20.6)
Sardines	9.8–13 (11.5 ± 1.7)	17–32 (21.5 ± 2.5)
Sand smelt	6.5–9 (7.72 ± 0.8)	4.2–6.5 (5.3 ± 0.6)

**Fig. 2:**
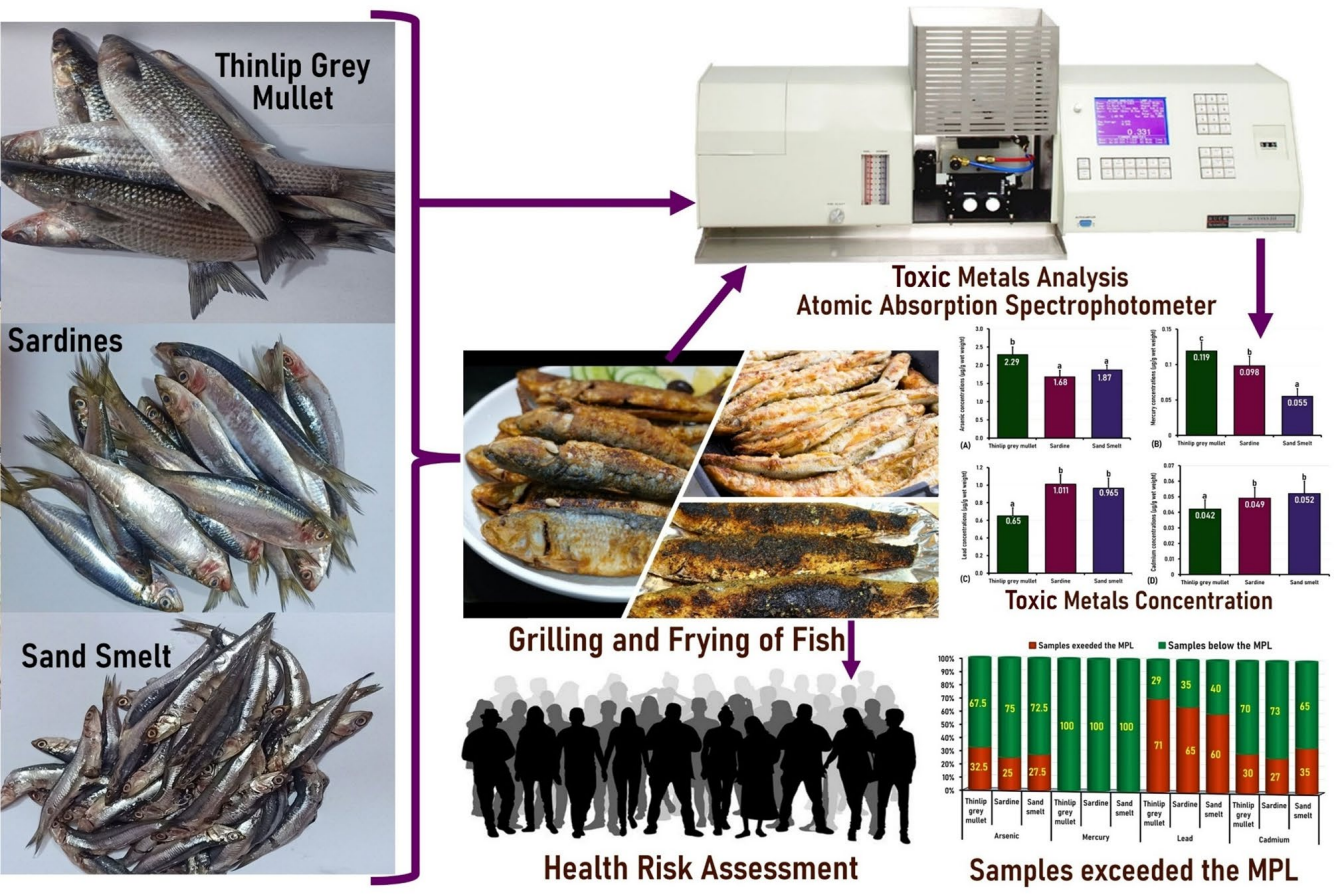
The graphical abstract summarizes the plan of the present study.

All methods were carried out in accordance with the guidelines and regulations of the Mansoura University-Research Ethics Committee, and all experimental protocols were approved by the Mansoura University Animal Care and Use Committee (MU-ACUC) under the proposal code of “VM.MS.24.12.202”, and the study is reported in accordance with ARRIVE guidelines. The graphical abstract summarized the plan of the present study is shown in Fig. 2.

### Reagent and washing procedure

Nitric acid (HNO_3_, ACS reagent ≥ 90.0%; Molecular Weight: 63.01) and perchloric acid (HClO_4_, ACS reagent 70%; molecular weight: 100.46) used for sample digestion were obtained from Merck (Merck KGaA, Darmstadt, Germany). All laboratory equipment used for sample storage and handling, such as glassware, tubes, and plastic items, was washed by soaking in water and soap for at least 2 h. After soaking, the wares were rinsed under running tap water multiple times, followed by a wash with deionized water. Next, they were rinsed with Therands mixture (250 mL deionized water + 200 mL conc. Hydrochloric acid “HCl” + 80 mL Hydrogen peroxide “H₂O₂”), followed by another rinse with washing acid (900 mL deionized water + 100 mL conc. HCl), and finally rinsed again with deionized water. The wares were kept dry on a clean bench until used.

### Sample preparation and digestion

Two grams of the dorsal muscle and the surrounding skin, starting just behind the nape and extending toward the fish’s tail, were aseptically weighed and cut with sterile stainless-steel scalpels and forceps from each sample. The muscle pieces were then macerated into small pieces using a ceramic knife before digestion. For the sample digestion, 2 g of each muscle sample were digested in a 20 mL screw-capped tube containing a mixture of 12 mL concentrated HNO_3_-HClO_4_ (comprising 8 mL conc. HNO_3_ and 4 mL conc. HClO_4_). The tubes were placed in a water bath at 53 °C and digested overnight^17^. This procedure yielded a final digest with a clear, colorless solution free of any visible residual organic material, indicating complete digestion of the samples. After cooling at room temperature, the digested samples were diluted with deionized water and filtered through Whatman Filter Paper Grade 42 (Merck KGaA; Darmstadt; Germany) into a clean glass beaker. The filtrate was brought to a final volume of 50 mL with deionized water.

Blank solutions without samples and standard solutions for each metal with pure certified metal were digested in the same method to define the background correction of the reagents, determine if chemicals and deionized water were contaminated with toxic metals, ensure the accuracy, and allow for deduction from the final results. The filtered samples were put at room temperature (25 °C) in a bottle that was cleaned, screw-capped, and labelled with a number and fish species until analysis could be conducted. Calibration curves were made with a stock solution of the analyzed metal with a concentration of 1000 mg/L diluted with ultrapure acidic water, 5% (v/v) HNO₃.

### Cooking methods (frying and grilling)

Ninety raw fish samples (30 each of the 3 fish species) were randomly selected to be subjected to either grilling (15 specimens from each fish species) or frying (15 specimens from each fish species). After cooling the cooked samples at ambient temperature, 2 g were taken from the same muscle on the opposite side of the fish and then prepared using the same procedures as those for the raw samples. Metal levels in the cooked fish were statistically compared with those in the corresponding raw samples.

### Toxic meal analysis

The filtered samples were analysed for As, Hg, Pb, and Cd (mg/kg wet weight) using the “Buck Scientific USA 210 VGP Atomic Absorption Spectrophotometer AAS”, which utilized an oxidizing air acetylene flame (Norwalk; CT; USA) for analysis of Pb and Cd, while the mercury hydride system (MHS) “cold vapour technique” was employed for determination of Hg and As.

The single hollow cathode lamp used in AAS serves as the line source. The AAS has a digital absorbance and concentration readout that can run based on specific instrumental guidelines^19^. The AAS-specific instrumental guidelines are presented in Table 2.

The following equation calculates the toxic metal values:

$${\text{Toxic metal content (mg/kg wet weight)}} = {\text{R}}\times {\text{D/W}}.$$

R is represented by the metal level reading (mg/L) obtained from AAS. D refers to the dilution factor of the prepared sample. W refers to the wet weight of the fish sample.

### Validation method

For calibration curve construction, standard solutions were prepared at the following concentrations: 0.01, 0.02, 0.1, 0.5, 5, and 20 mg/L for both Cd and As; 0.005, 0.01, 0.05, 0.25, 2.5, and 10 mg/L for Pb; and 0.01, 0.05, 0.1, 0.5, 2, and 5 mg/L for Hg. The validation method for measuring toxic metals was previously described^17,20^. The detection limits were determined at 0.01, 0.005, 0.02, and 0.005 mg/L, while the quantification limits were determined at 0.033, 0.017, 0.067, and 0.017 mg/L for As, Hg, Pb, and Cd, respectively. In this study, analytical blanks were used to correct for background contamination from reagents and the analytical procedure. The toxic metal concentration values in the blank samples were measured and subtracted from the sample readings to obtain the net concentrations of each sample analyzed. The precision percentages (repeatability; CV%) and spiking recovery percentages are shown in Table 2. To validate all analytical procedures used in the present study, a certified reference material (CRM) (Dogfish liver, DOLT-4) was obtained from the National Research Council of Canada (NRC-CNRC) (Table 2).

**Table 2:** Precision of the digestion method and certified reference material for heavy metal determination in fish samples.

Precision criteria		Toxic metals			
		As	Hg	Pb	Cd
AAS-specific instrumental guidelines	Lamp wavelengths (nm)	193.7	253.7	217.0	228.8
	Slit widths (nm)	0.7	0.2	0.7	0.7
	Lamp current (mA)	5	5	12	5
	Fuel (acetylene gas 99.9%) flow rates (L/min)	30	30	30	30
	Burner heights (cm)	8	7	8	8
Precision of Certified Reference Material (CRM)**	Certified value^1^ (mg/kg)	9.66 ± 0.62	2.58 ± 0.22	0.160 ± 0.04	24.3 ± 0.8
	Observed value^1^ (mg/kg)	10.04 ± 0.48	2.52 ± 0.19	0.157 ± 0.05	23.5 ± 0.7
	Recovery%	103.9	97.7	98.1	96.7
Precision of the digestion method for analyzing metals in the fish matrix (*n* = 5)	Concentration in fish sample (mg/kg)	2.12 ± 0.119	0.081 ± 0.006	0.85 ± 0.054	0.047 ± 0.007
	Amount of metal added (mg/kg)	5.00	0.500	2.00	0.250
	Concentration in the spiked sample (mg/kg)	6.99 ± 0.318	0.561 ± 0.022	2.69 ± 0.091	0.285 ± 0.019
	Recovery (%)	98.2%	96.6%	94.4%	96.6%
	RSD (%)	3.48%	4.12%	3.62%	3.98%

*The data are represented as the mean ± SE levels of triplicate analysis.

**DOLT-4: Dogfish liver certified reference material for trace metals (National Research Council of Canada; NRC - CNRC).

^1^Data represented by Mean ± SD; (*n* = 5).

### Health risk assessment

#### Estimated daily intake (EDIs)

For assessing non-carcinogenic risks through the consumption of tested fish, the EDI of As, Hg, Pb, and Cd was calculated according to (USEPA, 2000)^21^ through the following equation:

$${\text{EDI}}={\text{MC}} \times {\text{IR}}/{\text{BW}}$$

The EDI represents the estimated daily intake (mg/kg-d per day). IR refers to the daily ingestion rate of pelagic fish by an average 70 kg adult person per day in Egypt, which is 57.1 g (0.0571 kg/day) according to FAOSTAT^22^. MC refers to the levels of metals in tested fish samples (mg/kg wet weight). BW represents the average body weight of Egyptian consumers aged 16 to 70 years, which is 70 kg. The JECFA established the Benchmark Dose Levels (BMDL) for As at 3.0 µg/kg/day (3 × 10^−3^ mg/kg/day) and 1.6 µg/kg bw/week (1.6 × 10^−3^ mg/kg/week) for Hg, which is equivalent to 2.3 × 10^−4^ mg/kg/day^23^. The provisional tolerable monthly intake (PTMI) for Cd was set at 25 µg/kg bw/month (0.025 mg/kg bw/month), equivalent to 8.3 × 10^−4^ mg/kg/day^24^. Additionally, the European Food Safety Authority (EFSA) set the BMDL for Pb at 0.63 µg/kg/day (6.3 × 10^−4^ mg/kg/day)^25^.

#### Target hazard quotient (THQ)

The THQ is applied to assess probable non-carcinogenic health hazards associated with toxic metal exposure through the consumption of fish by adults over a lifetime. The hazard quotient was estimated based on the equation established by USEPA (2000)^21^:

$${\text{THQ}}={\text{EDI}}/{\text{RfD}}$$

The EDI represents the estimated daily intake of toxic metals (mg/kg/day). RfD refers to the oral reference doses, which are equivalent to 1 × 10^−4^ and 1 × 10^−3^ mg/kg/day for MeHg (methylmercury) and Cd, respectively^26^; 4 × 10^−3^ for Pb^27^; and 3 × 10^−4^ for As^28^. If THQ value > 1 indicates there are probable non-carcinogenic chronic public health risks^29^.

#### Hazard index (HI) or total target hazard quotient (TTHQ)

The HI or TTHQ is the sum of toxic metals from the consumption of tested fish. It evaluates non-carcinogenic public health hazards and is calculated using the equation provided by USEPA^30^:

$${\text{HI}}\left( {{\text{TTHQ}}} \right)={\text{THQ}}\left( {{\text{As}}} \right)+{\text{THQ }}\left( {{\text{Hg}}} \right)+{\text{THQ }}\left( {{\text{Pb}}} \right)+{\text{THQ }}\left( {{\text{Cd}}} \right).$$

If the HI (TTHQ) value exceeds 0.1, it indicates a non-carcinogenic health risk due to chronic toxicity.

#### Cancer risk (CR)

The CR is used to assess the cancer development associated with toxic metals-contaminated foods over a lifetime based on the following equation:

$${\text{CR}}={\text{EDI}} \times {\text{CSF}}$$

The EDI refers to the estimated daily intake of each toxic metal (mg/kg/day). CSF is the cancer slope factor (mg/kg/day) value of 1.5 and 0.38 for As and Cd, respectively^31^. No CSF value was recorded for Hg and pb. The CR value of 10^−4^ or above indicates a carcinogenic health risk. Conversely, if a CR value lies between 10^−6^ and 10^−4^, it is considered an acceptable limit and suggests no cancer risk^32^.

### Statistical analysis

All toxic metal measurements were carried out in triplicate. The mean standard error values ± (SE) were calculated. The data obtained was subjected to the one-way analysis of variance (ANOVA) for the differences in determining the toxic metal levels among the various fish species tested. Tukey’s honest significant difference test is used to assess the differences among the means of toxic metals for the three types of fish analyzed. Data were tested for normality (Shapiro–Wilk) and homogeneity of variances (Levene’s test). When assumptions were met, one-way ANOVA followed by Tukey’s HSD test was employed. When assumptions were violated, Welch’s ANOVA with Games–Howell post hoc correction was applied. The Student t-test was applied to detect the difference between raw and grilled fish, as well as between raw and fried ones. The significant difference was calculated at *P* < 0.05 or < 0.01. The data were analyzed using GraphPad PRISM^®^ 9.1.2 (GraphPad Software Incorporated, San Diego, USA) according to Sokal and Rohlf^33^.

## Results and discussion

### Toxic metal concentrations (mg/kg wet weight) in muscles of different fish species

Toxic metals are among the most dangerous substances, as they cannot be degraded by various biological systems and are bioaccumulated in the tissues of living organisms. Metals released into the aquatic environment may accumulate in the food chain, leading to environmental damage and posing a threat to human health, including cancer and damage to the cardiovascular, digestive, and nervous systems, as well as brain function. Arsenic is the most harmful metal, as even small amounts ingested over a long period can cause skin cancer and other serious health issues, including tiredness, sleeplessness, hair and weight loss^34^. Additionally, it is associated with various organ damage, carcinogenesis, and skin irritation^35^.

**Fig. 3:**
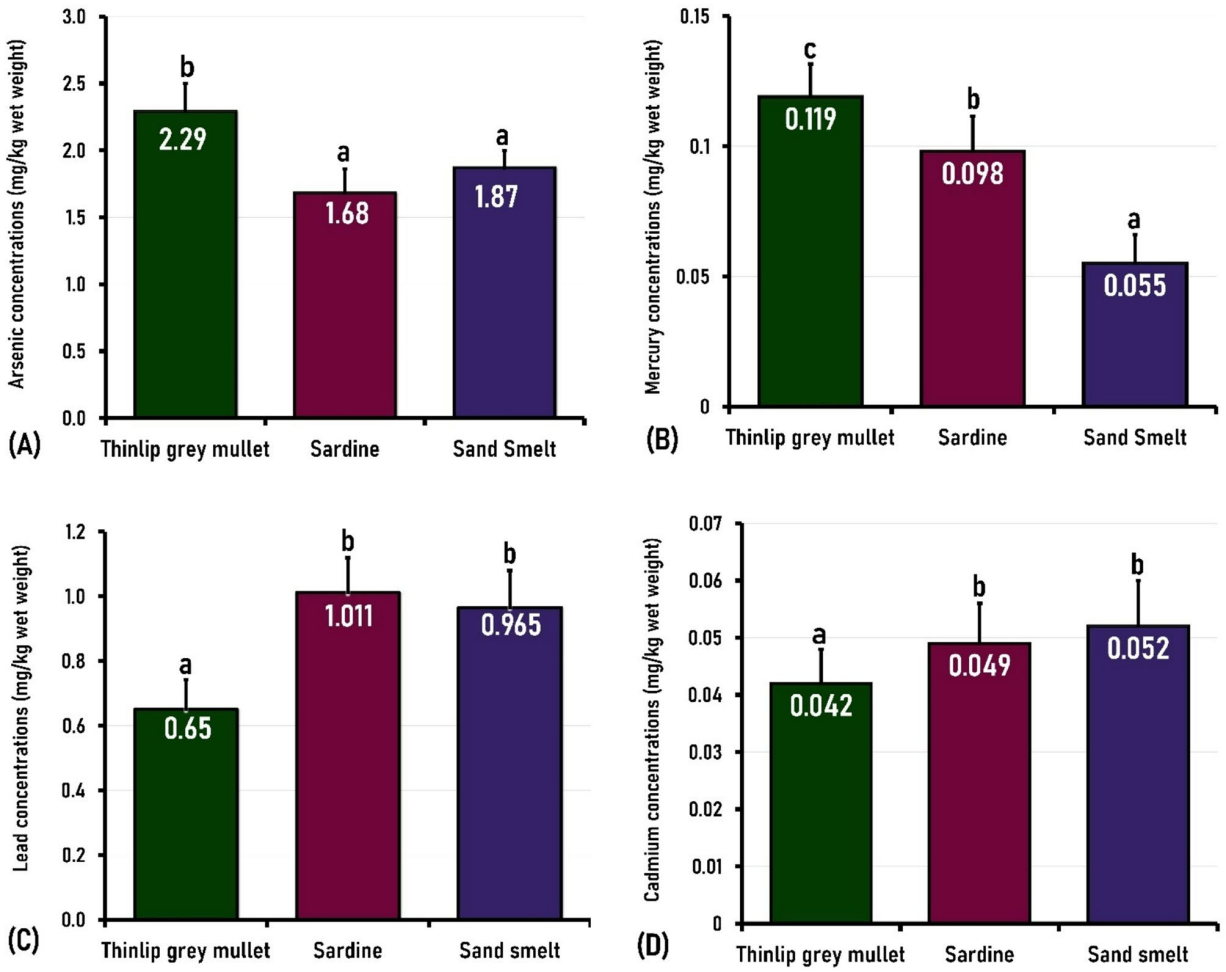
The mean concentrations of toxic metals (mg/kg wet weight) in thinlip grey mullet, European sardines, and sand smelt. Columns with different letters for each metal indicate significant differences (*P* < 0.01; or *P* < 0.05).

In this study, As levels in the fish samples tested ranged from 0.63 to 7.05 mg/kg, with a mean ± SE concentration of 2.29 ± 0.22 mg/kg for thinlip grey mullet; from 0.44 to 5.3 mg/kg, with a mean concentration of 1.68 ± 0.18 mg/kg for European sardine; and from 0.55 to 6.18 mg/kg, with a mean value of 1.87 ± 0.16 mg/kg for sand smelt (Table 3; Fig. 3). Fish species can be ordered from the highest to lowest levels of arsenic as: thinlip grey mullet > European sardine > sand smelt, with a significant difference (*P* < 0.05) in the As content between thinlip grey mullet and each of sardine and sand smelt (Fig. 3).

**Table 3:** Comparison of toxic metal concentrations in fish muscles (mg/kg wet weight) with the national and international maximum permissible limit (MPL).

Metals	MPL (mg/kg)	Thinlip grey mullet (*n* = 100)		Sardines (*n* = 100)		Sand smelt (*n* = 100)	
		Metal range (mg/kg)	Samples exceeded MPL	Metal range (mg/kg)	Samples exceeded MPL	Metal range (mg/kg)	Samples exceeded MPL
As	2^a^	0.63 − 7.05	32.5% (26/80)	0.44–5.3	25% (20/80)	0.55–6.18	27.5% (22/80)
Hg	0.5^b, c^	0.01–0.31	0% (0/80)	0.01–0.26	0% (0/80)	0.01–0.17	0% (0/80)
Pb	0.3^b, d^	0.01–2.22	70% (56/80)	0.01–2.38	65% (52/80)	0.03–2.19	60% (48/80)
Cd	0.05^b, e^	0.01–0.11	30% (24/80)	0.01–0.12	26.3% (21/80)	0.01–0.13	35% (28/80)

^a^Food Standards Australia New Zealand, 2020 (FSANZ, 2020)^36^.

^b^EOS (Egyptian Organization for Standards & Quality) (2010)^37^.

^c^Commission Regulation (EU) (2022) No 2022/617^38^.

^d^Commission Regulation (EU) (2021a) No 2021/1317^39^.

^e^Commission Regulation (EU) (2021b) No 2021/1323^40^.

Comparable As concentrations ranging from 3.53 to 3.94 mg/kg were reported in European sardines from the Western Mediterranean Sea, Spain^41^, as well as at a mean of 2.82 mg/kg in European sardines from the Adriatic Sea, Croatia^42^. On the other hand, arsenic was undetected in European sardines in many studies reported worldwide, including the Eastern Mediterranean Sea, Türkiye^43^, the Adriatic Sea, Italy^44^, and the Tyrrhenian Sea, Italy^45,46^. Moreover, the current results of As values in sand smelt was almost comparable to the levels of 2.19 ± 0.19 mg/kg in sand smelt caught from the Gulf of Patti, Italy^47^, even though it was 3 times higher than those demonstrated by Köker (2022)^48^, who reported As concentration ranging from 0.40 to 0.84 mg/kg with a mean of 0.63 ± 0.18 mg/kg in sand smelt caught from the Inzink lake basin, Türkiye.

Mercury is one of the most toxic metals that can enter aquatic environments through various means, such as coal-fired plants or industrial processes. Mercury toxicity can have significant effects on young children, resulting in learning disabilities, behavioural problems, and intellectual impairment^23^. In this study, Hg levels ranged from 0.01 to 0.31 mg/kg with a mean ± SE of 0.12 ± 0.02 mg/kg in thinlip grey mullet; 0.01 to 0.26 mg/kg with a mean ± SE level of 0.098 ± 0.02 mg/kg in European sardine; and 0.01 to 0.17 mg/kg with a mean ± SE level of 0.06 ± 0.06 mg/kg in sand smelt (Table 3; Fig. 3). Mercury concentrations in different fish species were ordered as thinlip grey mullet > European sardine > sand smelt, with a significant difference between the Hg level of thinlip grey mullet and each of sardine (*P* < 0.05) and sand smelt (*P* < 0.01); besides, there was a significant difference (*P* < 0.01) in Hg contents between sardine and sand smelt.

Our findings of Hg concentration are consistent with the level of 0.1 mg/kg reported in thinlip grey mullet from the Gironde estuary, France^49^, and with that reported in European sardines from the Western Mediterranean Sea, Spain, which ranged from 0.07 to 0.09 mg/kg^41^. In contrast, a lower Hg value of 0.05 mg/kg was detected in thinlip grey mullet samples caught from the Adriatic Sea, Italy^50^. On the other hand, Canli et al. (2001) found undetectable levels of Hg in European sardines from the Eastern Mediterranean Sea, Türkiye^43^, while higher Hg concentrations of 0.28 and 0.15 mg/kg were detected in sand smelt caught from the Inzink lake basin, Türkiye^48^, and that from the Axios Delta, Greece^51^.

Lead is recognized as a neurotoxic metal that leads to hepatic and renal damage, mental retardation, visual abnormalities, gingivitis, and nervous system damage, as well as irregularities in pregnancy and fertility, including spontaneous abortion^52^. Oil combustion, cigarette smoking, industrial waste, and vehicle exhaust are the primary sources of lead pollution^53^. In the current study, Pb was detected in all tested fish samples at concentrations ranging from 0.01 to 2.22 mg/kg, 0.01 to 2.38 mg/kg, and 0.03 to 2.19 mg/kg, with mean ± SE values of 10.65 ± 0.13 mg/kg, 1.011 ± 0.17 mg/kg, and 0.97 ± 0.19 mg/kg in thinlip grey mullet, European sardine, and sand smelt, respectively (Table 3; Fig. 3). The highest Pb level was observed in European sardine, while the lowest was found in thinlip grey mullet, allowing for their ranking as: sardine > sand smelt > thinlip grey mullet, with a significant (*P* < 0.01) difference between thinlip grey mullet and each of sardine and sand smelt (Fig. 2). The concentration of Pb may vary among different fish species even when caught from the same area due to several factors such as feeding level, fish size and age, foraging method, and the metal’s bioaccumulation capability in seafood^54^.

A higher mean Pb concentration of 1.88 mg/kg was detected in European sardines caught from the Latium coast, Italy^45^. Also, a high range of 1.160–1.680 mg/kg was determined in European sardines from the eastern Mediterranean Sea, Türkiye^43^. Lower Pb concentrations have been reported in other studies, including < 0.1 mg/kg in thinlip grey mullet from the Adriatic Sea, Montenegro^55^, 0.15 mg/kg in sardines from the Mediterranean Baltim coast, Egypt^56^, and 0.22 mg/kg in sand smelt from the Inzink Lake Basin, Türkiye^48^. Much lower Pb levels were reported for *Sardina pilchardus*, with 0.007 mg/kg in samples from the Mediterranean Sea, Italy^44^, and 0.002–0.05 mg/kg in those from the Mediterranean Sea, Morocco^57^. On the contrary, higher Pb levels of 6.12 and 5.57 were detected in sand smelt and *Sardines Pilchardus*, respectively, caught from the northeast Mediterranean Sea, Türkiye^58^. Additionally, an extremely high Pb level of 11.1 mg/kg (10 times higher than our findings) was detected in sardines marketed in Egypt^59^.

Cadmium is a toxic metal with no known biological function that can accumulate in the body, adversely affecting the kidneys, skeletal muscles, reproductive systems, and digestive systems^60^. In the present study, Cd levels ranged from 0.01 to 0.11, 0.01 to 0.12, and 0.01 to 0.13 mg/kg with mean ± SE values of 0.04 ± 0.01, 0.049 ± 0.01, and 0.06 ± 0.01 mg/kg, in thinlip grey mullet, sardine, and sand smelt, respectively (Table 3; Fig. 3). The highest Cd content was observed in sand smelt, while the lowest was in thinlip grey mullet. The order of mean Cd values, from highest to lowest, was as follows: sand smelt > European sardines > thinlip grey mullet, with significantly (*P* < 0.05) lower difference in Cd content between thilip grey mullet and each of sardine and sand smelt (Fig. 3).

Comparable mean Cd concentrations of 0.05 mg/kg^44^ and 0.03 mg/kg^48^ were detected in European sardines caught from the Mediterranean Sea, Italy, and in Sand smelt from the Inznik Lake Basin, Türkiye, respectively. Likewise, comparable Cd levels of 0.037, 0.04, and 0.05 mg/kg were reported in common sole, red porgy, and striped red mullet caught from the Mediterranean Sea coast in Egypt, respectively^61^. In contrast, lower Cd concentrations were reported, including 0.0019 mg/kg in thinlip grey mullet from the Adriatic Sea, Montenegro^55^, and 0.002–0.01 mg/kg in sardines marketed in Catalonia, Spain^41^. Higher Cd levels of 0.37 and 0.55 were found in sand smelt and *Sardines Pilchardus* caught from the northeast Mediterranean Sea, Türkiye^58^. The rate of toxic metal accumulation in different fish species varies based on the metal extracted, accumulation time, and the rate of scale formation^62^. Cadmium is primarily used in various industrial processes, such as zinc refining and lead smelting^63^.

These variations in heavy-metal levels among regions and species most likely reflect differences in local contamination sources, environmental conditions, and species ecology. Factors such as soil composition, water chemistry, industrial and urban discharges, and the degree of metal bioavailability in each aquatic system all have a significant impact on the accumulation of heavy metals in fish. The rate at which metals are bioaccumulated and biomagnified is also influenced by species-specific traits, including feeding patterns, trophic position, and habitat utilization.

### Fish acceptability based on the recommended maximal permissible limits (MPLs)

**Fig. 4:**
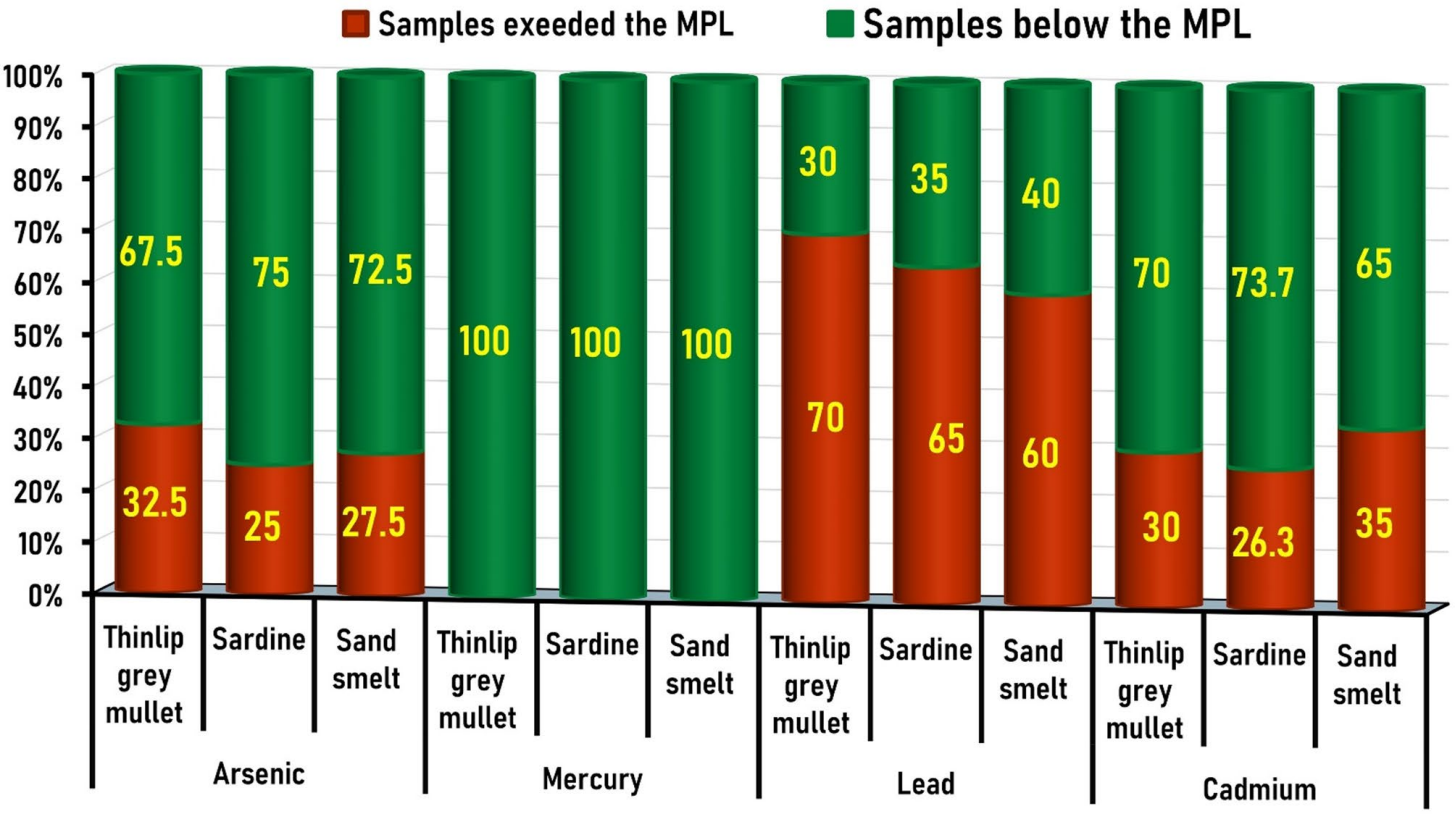
Fish acceptability based on the recommended maximum permissible limits (MPLs) showed the percentage of fish samples exceeded the MPLs versus those within the MPLs for toxic metals analyzed in thinlip grey mullet, European sardines, and sand smelt.

The present results indicated that 32.5% (26/80), 25% (20/80), and 27.5% (22/80) of the tested thinlip grey mullet, sardines, and sand smelt, respectively, contained As levels higher than the proposed legal limit^36^ (Table 3; Fig. 4). The elevated levels of As in more than 28.3% of fish samples may reflect industrial discharge and untreated domestic effluents near Damietta.

On the other hand, Sabala et al. (2024) found that 100% of sardines marketed in Egypt exceeded the recommended limits for As^64^. Likewise, Mahmoud et al. (2025) revealed that all (100%) of the marine fish samples from another species collected from the Mediterranean coast at Damietta City, Egypt, exceeded the MPL set for As^61^.

Interestingly, Hg contents in the current study were detected in all fish samples at low concentrations, remaining below the MPL of 0.5 mg/kg set by EOS (2010)^37^ as well as by the Commission Regulation (EU) (2022)^38^. Similarly, the Hg levels in all (100%) sardine samples marketed in Egypt were within the safe limits^64^. On the other hand, only 12% (12/100) of sardine samples caught from the Mediterranean Sea in Egypt exceeded the MPL for Hg^56^.

The percentages of fish samples that exceeded the Pb permissible limit (0.3 mg/kg)^37,39^. in this study were 70% (56/80), 65% (52/80), and 60% (48/80) of thinlip grey mullet, sardines, and sand smelt, respectively. Previous studies in Egypt reported that the Pb contents exceeded the recommended limits in 45% (36/80)^64^ and 10% (10/100)^56^ of examined sardine samples. Concerning Cd tested in this study, 30% (24/80) of thinlip grey mullet, 26.3% (21/80) of sardines, and 35% (28/80) of sand smelt exceeded the permissible Cd limit of 0.05 mg/kg^37,40^. A previous study in Egypt reported that 90% (72/80) of sardine samples exceeded the recommended Cd limits^64^, while another study found that only 6% (6/100) of sardine samples surpassed these limits^56^.

### Effect of cooking on metal contents in fish samples tested

In this study, frying of thinlip grey mullet samples exhibited reduction percentages of 45.6%, 66.7%, 36.3%, and 75% in As, Hg, Pb, and Cd levels, respectively, with significant differences observed in all metal levels before and after frying (Table 4). In sardine samples, frying resulted in a reduction of 10.71% for As, 83.33% for Hg, 30.16% for Pb, and 60% for Cd, while in sand smelt, frying exhibited a reduction of 16.33%, 37.5%, 63.87%, and 75% in As, Hg, Pb, and Cd levels, respectively, with significant differences (*P* < 0.05) in Hg, Pb, and Cd levels before and after frying (Table 4). Significant pre- and post-frying differences were observed for Hg, Pb, and Cd in the three fish species tested, whereas As did not show a significant (*P* > 0.05) change in sardine and sand smelt, although it demonstrated a pre- and post-frying significant change in thinlip grey mullet (Table 4). The reduction in metal values resulting from cooking methods may be attributed to their release through drips as free salts, possibly in connection with soluble amino acids and uncoagulated proteins^65^, as well as cooking decreasing the fish protein content^66^.

**Table 4:** Effect of cooking on toxic metal levels in thinlip grey mullet, sardine, and sand smelt.

Fish spp.	Cooking method	Metals	Metal levels (mg/kg)		Reduction%	*P*
			Before	After		
Thinlip grey mullet	Frying	As	2.63 ± 0.23^a^	1.43 ± 0.15^b^	45.62%	*
		Hg	0.15 ± 0.02^a^	0.05 ± 0.004^b^	66.67%	**
		Pb	0.91 ± 0.08^a^	0.58 ± 0.04^b^	36.26%	*
		Cd	0.04 ± 0.003^a^	0.01 ± 0.001^b^	75%	**
	Grilling	As	1.97 ± 0.17^a^	1.27 ± 0.011^b^	35.53%	*
		Hg	0.09 ± 0.007^a^	0.06 ± 0.004^b^	33.33%	*
		Pb	0.63 ± 0.05 ^a^	0.53 ± 0.03^a^	15.87%	–
		Cd	0.05 ± 0.003^a^	0.030 ± 002^b^	40%	*
Sardine	Frying	As	2.24 ± 0.19^a^	2.0 ± 0.14^a^	10.71%	–
		Hg	0.12 ± 0.011^a^	0.02 ± 0.001^b^	83.33%	**
		Pb	1.26 ± 0.13^a^	0.88 ± 0.06^b^	30.16%	*
		Cd	0.05 ± 0.004^a^	0.02 ± 0.001^b^	60%	**
	Grilling	As	1.76 ± 0.15^a^	1.58 ± 0.13^a^	10.23%	–
		Hg	0.15 ± 0.011^a^	0.1 ± 0.008^b^	33.33%	*
		Pb	1.2 ± 0.10^a^	0.43 ± 0.03^b^	64.17%	**
		Cd	0.03 ± 0.002^a^	0.02 ± 0.001^b^	33.33%	*
Sand smelt	Frying	As	2.45 ± 0.21^a^	2.05 ± 0.16^a^	16.33%	–
		Hg	0.08 ± 0.006^a^	0.05 ± 0.003^b^	37.5%	*
		Pb	1.19 ± 0.09^a^	0.43 ± 0.03^b^	63.87%	**
		Cd	0.04 ± 0.003^a^	0.01 ± 0.001^b^	75%	**
	Grilling	As	2.29 ± 0.18^a^	1.62 ± 0.13^a^	29.23%	–
		Hg	0.06 ± 0.004^a^	0.02 ± 0.002^b^	66.67%	**
		Pb	0.96 ± 0.07^a^	0.74 ± 0.05^a^	22.92%	–
		Cd	0.05 ± 0.003^a^	0.02 ± 0.001^b^	60%	**

^a,b^Values (mean ± SE) with different superscript letters are significantly different.

*P* < 0.05 (*); *P* < 0.01(**); (-): *P* > 0.05.

Similar to our results, roasting has been reported to decline Hg levels by 63% in sardine samples^67^. In contrast, pan-frying elevated the concentrations of Hg, Pb, and Cd by 377%, > 200%, and 223%, in shrimp samples, respectively^68^. Additionally, the Cd level increased by 250% in cooked female crabs and 100% in cooked male crab flesh^69^. Moreover, frying raised the Cd content in shrimps by 55% and the Pb level in lobster by 29%^70^. This increase in metal content during frying might be linked to the evaporation processes involved during frying.

Grilling of thinlip grey mullet resulted in a reduction of As, Hg, Pb, and Cd residues by 35.53%, 33.33%, 15.87%, and 40% with a significant (*P* < 0.05) difference detected before and after grilling for As, Hg, and Cd but not for Pb (Table 4). Grilling of sardine, however, resulted in 10.23%, 33.33%, 64.17%, and 33.33% reductions in As, Hg, Pb, and Cd contents, respectively in sardine with a significant (*P* < 0.05) difference in the metals analyzed, except for As, along with reduction by 29.23%, 66.67%, 22.92%, and 60%, respectively in sand smelt, with a significant difference detected before and after grilling in Hg and Cd, but without a substantial difference in As and Pb in sand smelt (Table 4).

Grilling has been reported to produce controversial effects in different fish species tested worldwide. In Egypt, grilling produced a significant reduction in Hg, As, Pb, and Cd contents in common sole and red porgy^61^, although it didn’t exhibit a significant reduction in the mean levels of metals (As, Hg, Pb, Cd) tested, except for Pb in crabs and Cd in shrimp samples^71^. Conversely, in Spain, Devesa et al. (2001)^72^ demonstrated that grilling increased As contents in marketed bivalves and squid fish. In another study, Tawfik (2013) observed a 218% increase in Cd content in boiled shrimp from Saudi Arabia^73^, whereas Gheisari et al. in Iran reported that boiling reduced Pb content by 13% in lobster and 35% in shrimp flesh caught from the Persian Gulf^70^.

Upon comparing the mean reduction percentages of the measured toxic metals (As, Hg, Pb, and Cd) between cooking methods, frying consistently showed higher removal efficiency than grilling across fish species. In thinlip grey mullet, frying achieved an average reduction of 55.9%, significantly higher than the 31.2% observed with grilling. A similar pattern was noted in sardine, where frying reduced metal levels by 46.1% compared with 35.3% for grilling. In sand smelt, both methods produced comparable results; however, frying still showed a marginally higher reduction (48.2%) compared with grilling (44.7%). Overall, these findings indicate that frying is generally more effective than grilling in decreasing heavy-metal residues in fish, particularly in species with higher initial contamination levels.

### Health risk assessment associated with the consumption of toxic metals-contaminated fish

The toxicological reference values adopted in this study were selected from internationally recognized risk-assessment authorities, namely the USEPA, JECFA, and EFSA. These organizations routinely establish health-based guidance values based on comprehensive toxicological datasets, standardized evaluation procedures, and transparent uncertainty analyses. Despite having similar goals, these organizations differ in several methodological aspects^74,75^. The USEPA primarily provides reference doses (RfDs) and cancer slope factors (CSF) that are based on United States-specific exposure scenarios and often focus on chronic daily exposure limits. JECFA, which operates under WHO and FAO, evaluates contaminants from a universal dietary perspective and typically expresses guidance values as provisional tolerable weekly or monthly intakes (PTWI/PMTIs). EFSA, meanwhile, applies European population exposure data and may adopt more conservative limits due to thorough reassessment processes and frequent updates of scientific opinions. These differences among the organizations reflect the mandatory regulations and population focus of each agency^76,77^. Therefore, in this study, the most suitable and generally accepted guidance value for each pollutant was selected from among these sources to ensure a reliable, transparent, and internationally comparable health-risk assessment.

In the present study, non-carcinogenic and carcinogenic public health risks associated with consuming metal-contaminated fish were assessed. The estimated daily intakes (EDIs), Target Hazard Quotients (THQs), and Total Target Hazard Quotients (TTHQs) were adopted for non-carcinogenic assessment^17,20,29,32^, while the Target Cancer Risk (TCR) was applied for carcinogenic assessment from the consumption of metal-contaminated fish over a lifetime^31^.

#### Estimated daily intake (EDI) of detected metals in examined fish samples in comparison to their provisional tolerable daily intake (PTDI)

The EDI values represent the safe levels of toxic metals, which are used to assess the non-carcinogenic risk associated with consuming metal-contaminated fish. According to the Food and Agriculture Organization^22^, the average ingestion rate of fish for an adult typically consuming, weighing 70 kg, is 57.096 g/day.

Comparing the EDI values of As, Hg, and Cd from the consumption of thinlip grey mullet, sardines, and sand smelt samples tested in the present study with their PTDI or BMDL (EDI/PTDI or BMDL) set by JECFA (2010a, b)^23,24^ and EFSA (2010)^25^ revealed low percentages (< 100%), which indicated non-carcinogenic risks from these three metals through the consumption of the three fish species tested (Table 5). On the other hand, the EDI of Pb exceeded the PTDI by 130.8% and 124.9% through consumption of sardine and sand smelt, respectively, while the EDI to PTDI ratio of Pb was 84.29% through consumption of thinlip grey mullet samples (Table 5), revealing that there may be a probable non-carcinogenic risks from Pb through the consumption of sardine and sand smelt but not from thinlip grey mullet.

**Table 5:** Toxic metal concentrations in the muscles of sampled thinlip grey mullet, sardine, and sand smelt fish and their estimated daily intake (EDI) compared to either their provisional tolerated daily intake (PTDI) or their benchmark dose levels (BMDL).

Toxic metals	PTDI/BMDL (mg/kg bw/day)	Mean concentration of toxic metals (mg/kg)			EDI (mg of metal/70-kg BW person/day) in comparison with PTDI or BMDL					
		Thinlip grey mullet	Sardine	Sand smelt	Thinlip grey mullet		Sardine		Sand smelt	
					EDI (mg/kg bw/d)	%EDI/PTDI or BMDL	EDI (mg/kg bw/d)	%EDI/PTDI or BMDL	EDI (mg/person/d)	%EDI/PTDI or BMDL
As	3.00 × 10^−3a^	2.29	1.68	1.87	1.87 × 10^−3^	62.33%	1.37 × 10^−3^	45.67%	1.53 × 10^−3^	50.84%
Hg	2.30 × 10^−4a^	0.1195	0.098	0.0545	9.75 × 10^−5^	42.39%	7.99 × 10^−5^	34.75%	4.45 × 10^−5^	19.33%
Pb	6.30 × 10^−4b^	0.651	1.0105	0.9645	5.31 × 10^−4^	84.29%	8.24 × 10^−4^	130.83%	7.87 × 10^−4^	124.87%
Cd	8.30 × 10^−4c^	0.042	0.0485	0.0515	3.43 × 10^−5^	4.13%	3.96 × 10^−5^	4.77%	4.20 × 10^−5^	5.06%

^a^JECFA (2010a)^23^.

^b^EFSA (2010)^25^.

^c^JECFA (2010b)^24^.

$$\:\text{E}\text{D}\text{I}\:=\frac{\text{M}\text{C}\:\times\:\:\text{I}\text{R}}{\text{B}\text{W}}$$.

MC is the metal concentration mean in sampled fish, expressed as mg/kg wet weight.

IR is a daily ingestion rate for an adult typically consumes 57.096 g (57.096 × 10^−3^ Kg) of fish per day according to FAO (2022)^22^.

BW is 70 kg, an average weight for the Egyptian fish eaters aged between 16 and 70 years.

The formula for calculating the Estimated Daily Intake (EDI) of toxic metals EDI (mg kg^−^¹ bw day^−^¹) set by USEPA (2000)^21^.

The present results of the EDI values are in agreement with those reported in Türkiye^78^. where the EDI values for Cd and Pb from the consumption of thinlip grey mullet were below the recommended PTWI levels set by FAO/WHO^79^. Similarly, the EDI values for Pb through consumption of thinlip grey mullet in Egypt were also below the acceptable limits^80^. Moreover, the EDI values for As, Hg, Pb, and Cd analyzed for the Egyptian population through the consumption of sardines were under their PTDI/BMDL^64^. Additionally, Falcó et al. (2006) found daily intake of As, Hg, Cd, and Pb by the Catalonia population was under the respective provisional tolerable weekly intake (PTWI) values through the consumption of fish and seafood^41^. In contrast, Türkmen et al.^74^ in Türkiye indicated that the EDI values for Cd from the consumption of *C*. *Ramada* exceeded the limits set by FAO/WHO^75^.

It should be noted that the consumption rate (57 g/day) in this study reflects the average intake for normal adults and may underestimate exposure in vulnerable or high-consumption groups. This value, however, may underestimate exposure for vulnerable or high-consumption subgroups such as children, pregnant women, fishermen, and coastal communities. These groups may ingest proportionally higher amounts of fish relative to their body weight, resulting in higher estimated daily intake (EDI) and consequently higher hazard quotients or cancer risks. Consequently, the present risk estimates likely represent conservative estimates for these groups, and future assessments should incorporate subgroup-specific or upper-percentile consumption scenarios to more accurately characterize risk among these vulnerable populations.

#### The THQs and TTHQs of toxic metals detected in fish species for assessment of non-carcinogenic risks in the Egyptian population

The THQ and TTHQs (HI) are methods used to assess non-carcinogenic health risks associated with consuming fish contaminated by metals. The TTHQ value represents the sum of the THQ values of all toxic metals tested in each fish species. A THQ or TTHQ value of less than one indicates no non-carcinogenic health risk from consuming fish contaminated by metals. However, if the THQ or TTHQ value exceeds one, it suggests a potential non-carcinogenic public health risk^29^.

The THQ values of toxic metals tested due to the consumption of thinlip grey mullet, sardines, and sand smelt by the Egyptian population are listed in Table 6. The THQ values of As in all examined fish samples were above 1, suggesting that there were non-carcinogenic risks from consuming these fish species harvested from the Mediterranean Sea at the Damietta Coast. On the contrary, the THQ values for Hg, Pb, and Cd were all below 1, indicating that these metals do not pose non-carcinogenic public health risks from the consumption of these fish species.

**Table 6:** Target hazard quotient (THQ) and hazard index (HI) or total target hazard quotient (TTHQ) of toxic metals resulting from consumption of thinlip grey mullet, sardine, and sand smelt.

Metals	RfD (mg/kg/day)	Thinlip grey mullet		Sardine		Sand smelt	
		EDI (mg/kg BW/day)	THQ	EDI (mg/kg BW/day)	THQ	EDI (mg /kg BW/day)	THQ
As	3 × 10^−4b^	1.87 × 10^−3^	6.23	1.37 × 10^−3^	4.57	1.53 × 10^−3^	5.1
Hg	1 × 10^−4a^	9.75 × 10^−5^	0.975	7.99 × 10^−5^	0.80	4.45 × 10^−5^	0.445
Pb	4 × 10^−3c^	5.31 × 10^−4^	0.133	8.24 × 10^−4^	0.21	7.87 × 10^−4^	0.197
Cd	1 × 10^−3a^	3.43 × 10^−5^	0.034	3.96 × 10^−5^	0.040	4.20 × 10^−5^	0.042
Σ HI or TTHQ^d^			7.37		4.62		5.78

EDI refers to estimated daily intake.

BW refers to body weight.

$$\:THQ=\frac{EDI}{RfD}$$.

RfD refers to the oral reference dose (mg/kg/day).

^a^RfD for Hg (MeHg) and Cd (USEPA, 2019)^26^.

^b^RfD for As (ATSDR, 2007)^28^.

^c^RfD for Pb (FDA, 2018)^27^.

^d^Hazard Index (HI) or Total Target Hazard Quotient (TTQH) = THQAs + THQHg + THQPb + THQCd (USEPA, 2011)^81^.

Our results of THQ are consistent with those of Cd and Pb, which were below one from consuming thinlip grey mullet caught from the Adriatic Sea, Montenegro, indicating no adverse effects on lifetime intake of these metals^55^. Similarly, the THQ of Cd and Hg in sardines caught from the Algerian coast of the Mediterranean Sea were lower than one^82^. In contrast, the THQ value of As from the consumption of sardines obtained from Italian supermarkets exceeded one^11^. Furthermore, the THQ values of Pb and Cd in thinlip mullet collected from freshwater, brackish, and offshore sites varying in salinity in Egypt were above one, suggesting that consumers may face some adverse health effects from these metals when consuming these fish^76^.

The TTHQ values for the four tested metals in each fish species were greater than one (7.37, 4.62, and 5.78 for thinlip grey mullet, sardines, and sand smelt, respectively), indicating a potential non-carcinogenic risk for individuals consuming these fish from the Mediterranean Sea at the Damietta coast among the Egyptian population. Our results align with those reported by Kasmi et al. (2023), who identified TTHQ values exceeding one from the intake of *Sardina pilchardus* from the Moroccan Mediterranean Coast, suggesting non-carcinogenic risks to public health^57^. In contrast, Antović et al. (2019) found that the TTHQ value from consuming *C*. *ramada* was less than one, indicating that this fish could be safely consumed^55^. Additionally, the TTHQ or HI value was determined below one from consuming sardines harvested from the Mediterranean Sea, at Baltim coast, Egypt^56^.

#### Cancer risk (CR) assessment in the Egyptian population through consumption of toxic metal-contaminated fish

The CR is a method used to detect carcinogenic risks from consuming food contaminated with toxic metals; if the CR exceeds 10^−4^, carcinogenic risks are likely. In this study, the CR values for As in all tested fish species surpassed 1.0 × 10^−4^ (Table 7), indicating potential carcinogenic risks associated with consuming these fish. Meanwhile, the CR values for Cd in all tested fish samples were below 1.0 × 10^−4^, suggesting that consuming fish with these metals is unlikely to pose carcinogenic risks to consumers (Table 7).

Similar to the present results, the carcinogenic risk value for As in *Sardina pilchardus* marketed in Egypt was above the acceptable limits^64^. Additionally, indicated that the CR for Hg, As, Pb, and Cd in *Sardina pilchardus* from the Mediterranean Coast, Morocco, was above the recommended limits, resulting in a potential carcinogenic risk to human health^57^.

Because this study quantified only total arsenic, the resulting risk assessment (THQ and CR) may not accurately reflect exposure to inorganic arsenic (iAs), which is the toxicologically active species. Therefore, incorporation of comprehensive arsenic speciation analyses in future investigations is warranted to provide more accurate and reliable risk estimates.

**Table 7:** The cancer risk (CR) associated with the consumption of thinlip grey mullet, sardine, and sand smelt fish samples contaminated with toxic metals, predicated on the estimated daily intake (EDI) (mg/kg/day) and cancer slope factor (CSF) (mg/kg/day).

Metals	*CSF (mg/kg bw/day)	Thinlip grey mullet		Sardine		Sand smelt	
		EDI (mg/kg bw/d)	CR	EDI (mg/kg bw/d)	CR	EDI (mg/kg bw/d)	CR
As	1.5	1.87 × 10^−3^	2.81 × 10^−3^	1.37 × 10^−3^	2.1 × 10^−3^	1.53 × 10^−3^	2.3 × 10^−3^
Hg**	–	9.75 × 10^−5^	–	7.99 × 10^−5^	–	4.45 × 10^−5^	–
Pb**	–	5.31 × 10^−4^	–	8.24 × 10^−4^	–	7.87 × 10^−4^	–
Cd	0.0018	3.43 × 10^−5^	6.17 × 10^−8^	3.96 × 10^−5^	7.12 × 10^−8^	4.20 × 10^−5^	7.56 × 10^−8^

$$CR=CSF\times EDI.$$

Danger exceeding 10⁻^4^ suggests carcinogenic danger, but risk between 10⁻^6^ and 10⁻^4^ is acceptable.

The cancer risk equation is mentioned by USEPA (2024).

*The value of CSF for metals tested was defined by USEPA (2024).

**The U.S. EPA does not have a cancer slope factor (CSF) for mercury and lead in food because it is not classified as a carcinogen by the agency.

## Conclusion

The study concluded that a significant number of fish samples examined had toxic metal levels exceeding their MPLs. However, thermal processing methods such as frying and grilling were found to reduce the average concentrations of toxic metals in the tested fish. Although the EDI values for As, Hg, and Cd analyzed in three fish species were lower than the PTDI set by many organizations, they surpassed the PTDI for Pb in the tested sardines and sand smelt, indicating a possible non-carcinogenic hazard from Pb through consumption of these fish species. Furthermore, the calculated THQ, TTHQ, and CR values indicated potential human health risks from arsenic exposure through consumption of the three fish species tested, highlighting the need for risk management measures. These measures include issuing evidence-based consumption advisories, implementing stricter control of contamination sources, enhancing routine monitoring of aquatic environments and fish species, and increasing public awareness to ensure a long-term reduction of human exposure.

Finally, the results demonstrate that As and Pb are the main contaminants of concern in fish from the Damietta coast, and although cooking methods reduced metal concentrations to some extent, the carcinogenic risk associated with As remained above acceptable limits. These findings call for stricter environmental monitoring and evidence-based fish consumption advisories for local populations.

## Data Availability

The data sets generated in this study are included within the article.
